# Mass spectrometry-based serum proteome pattern analysis in molecular diagnostics of early stage breast cancer

**DOI:** 10.1186/1479-5876-7-60

**Published:** 2009-07-13

**Authors:** Monika Pietrowska, Lukasz Marczak, Joanna Polanska, Katarzyna Behrendt, Elzbieta Nowicka, Anna Walaszczyk, Aleksandra Chmura, Regina Deja, Maciej Stobiecki, Andrzej Polanski, Rafal Tarnawski, Piotr Widlak

**Affiliations:** 1 Maria Skłodowska-Curie Memorial Cancer Center and Institute of Oncology, Gliwice, Poland; 2 Polish Academy of Science, Institute of Bioorganic Chemistry, Poznan, Poland; 3 Silesian University of Technology, Gliwice, Poland; 4 Polish-Japanese Institute of Information Technology, Bytom, Poland

## Abstract

**Background:**

Mass spectrometric analysis of the blood proteome is an emerging method of clinical proteomics. The approach exploiting multi-protein/peptide sets (fingerprints) detected by mass spectrometry that reflect overall features of a specimen's proteome, termed proteome pattern analysis, have been already shown in several studies to have applicability in cancer diagnostics. We aimed to identify serum proteome patterns specific for early stage breast cancer patients using MALDI-ToF mass spectrometry.

**Methods:**

Blood samples were collected before the start of therapy in a group of 92 patients diagnosed at stages I and II of the disease, and in a group of age-matched healthy controls (104 women). Serum specimens were purified and the low-molecular-weight proteome fraction was examined using MALDI-ToF mass spectrometry after removal of albumin and other high-molecular-weight serum proteins. Protein ions registered in a mass range between 2,000 and 10,000 Da were analyzed using a new bioinformatic tool created in our group, which included modeling spectra as a sum of Gaussian bell-shaped curves.

**Results:**

We have identified features of serum proteome patterns that were significantly different between blood samples of healthy individuals and early stage breast cancer patients. The classifier built of three spectral components that differentiated controls and cancer patients had 83% sensitivity and 85% specificity. Spectral components (i.e., protein ions) that were the most frequent in such classifiers had approximate m/z values of 2303, 2866 and 3579 Da (a biomarker built from these three components showed 88% sensitivity and 78% specificity). Of note, we did not find a significant correlation between features of serum proteome patterns and established prognostic or predictive factors like tumor size, nodal involvement, histopathological grade, estrogen and progesterone receptor expression. In addition, we observed a significantly (p = 0.0003) increased level of osteopontin in blood of the group of cancer patients studied (however, the plasma level of osteopontin classified cancer samples with 88% sensitivity but only 28% specificity).

**Conclusion:**

MALDI-ToF spectrometry of serum has an obvious potential to differentiate samples between early breast cancer patients and healthy controls. Importantly, a classifier built on MS-based serum proteome patterns outperforms available protein biomarkers analyzed in blood by immunoassays.

## Background

In recent years cancer diagnostics has been taking enormous advantage of genomics and proteomics, novel fields of modern biology. Proteomics is the study of the proteome, the complete protein components of the cell, tissue or organism, which in contrast to the genome is dynamic and fluctuates depending on a combination of numerous internal and external factors (e.g., physiological status, dietary behavior, stress, disease and medical treatment). Identifying and understanding changes in the proteome related to disease development and therapy progression is the subject of clinical/disease proteomics [1,2]. It is currently well appreciated that because of the complexity of molecular processes involved in cancer no particular molecular feature alone, neither gene nor protein, could be a reliable biomarker in cancer diagnosis. Instead, multi-component molecular classifiers, exemplified by multi-gene cancer signatures implemented in the functional genomics field, are built and successfully applied. Multi-gene signatures identified for breast cancer have proved their diagnostic power even though detailed knowledge about the function of particular genes that build such signatures may not be available at present [3,4].

The low molecular weight (<10 kDa) component of the blood proteome is a promising source of previously undiscovered biomarkers. Since this protein fraction is below the limit of effective resolution of conventional gel electrophoresis, mass spectrometric analysis appears to be a method of choice [5], and consequently is an emerging method of clinical proteomics and cancer diagnostics [rev. in: [6-9]]. The milestone paper in this field was published in 2002 by the group of Petricoin and Liotta, who showed that components of the serum proteome identified by mass spectrometry differentiate patients with ovarian cancer from healthy individuals [10]. Since that time, in spite of a certain controversy regarding this pioneering work [11], numerous papers have been published that aimed to verify the applicability of mass spectrometric analyses of the serum (or plasma) proteome for cancer diagnostics. Although no single peptide could be expected to be a reliable bio-marker in such analyses, multi-peptide sets of markers selected in numerical tests have been shown already in a few studies to have potential prognostic and predictive values for cancer diagnostics [rev. in: [12-16]]. The approach that takes into consideration features of the whole proteome, e.g. protein fingerprints given by mass spectra or 2D gel electrophoresis but does not rely on particular identified protein(s), could be called proteome pattern analysis or proteome profiling. In this approach, whose strategy is similar to the search for multi-gene signatures in functional genomics, multi-component sets of peptides/proteins (which are exemplified by ions registered at defined m/z values in the mass spectrum) define specific proteomic patterns (or profiles), allowing one to classify samples even though their particular components lack differentiating power when analyzed separately. Importantly, such pattern/profile reflects features of the specimen's proteome and allows its classification even without detailed knowledge about particular elements [17-19]. Mass spectrometry methods particularly suitable for proteome pattern analysis are Matrix-Assisted Laser Desorption-Ionization spectrometry (MALDI) and its derivative Surface-Enhanced Laser Desorption/Ionization spectrometry (SELDI) coupled to a Time-of-Flight (ToF) analyzer, which combine high throughput, fair sensitivity and accuracy of annotation of m/z values of ions in recorded mass spectra of complex protein mixtures such as biological specimens [20,21]. The relevance of mass spectrometry-based serum (or plasma) proteome pattern analysis has been already tested for several type of human malignancies though none of identified peptide signatures was approved for diagnostics in clinical practice, as yet [15,22-26].

Breast cancer is the most common malignancy in women, comprising about 18% of all female cancers, and 1 million new cases occur worldwide each year. In Western countries the disease is the single commonest cause of death among women aged 40–50, accounting for about a fifth of all deaths in this age group [27]. The most important tools in screening and early detection of breast cancer are imaging techniques: mammography, ultrasonography and magnetic resonance imaging. Unfortunately however, up to 20% of new breast cancer incidents cannot be detected by these methods [28], indicating a constant need for novel molecular markers suitable for screening and early detection of this cancer. Several studies have already addressed the possibility of applying SELDI or MALDI mass spectrometric analyses of blood proteome in diagnostics of breast cancer, and elicited serum (or plasma) proteome patterns specific for patients with breast cancer at either early or late clinical stages [29-38]. Among the peptides identified in such differentiating patterns were fragments of C3a [33] and of FPA, fibrinogen, C3f, C4a, ITIH4, apoA-IV, bradykinin, factor XIIIa and transthyrein [35]. In addition, mass spectrometry analyses of the blood proteome allowed the identification of patterns specific for breast cancer patients with different outcome and response to therapy [39-43]. Different methodological approaches, both experimental and computational, have been implemented in such studies, and the proposed proteome patterns specific for breast cancer consisted of different peptide sets. However, several peptides that differentiated cancer and control samples appeared reproducibly when comparative analysis across different studies was performed [44], demonstrating the high potential of mass spectrometry-based analyses of the blood proteome pattern in diagnostics of breast cancer once problems with standardization of experimental and computational design are solved.

Here we examined the potential applicability of the serum proteome pattern identified by MALDI-ToF mass spectrometry, either alone or in combination with protein biomarkers analyzed by immunoassays, in early detection of breast cancer. The spectral components that were annotated on the basis of recorded mass spectra were successfully used to build classifiers that allowed reliable identification of early stage breast cancer patients. Importantly, the classifier based on serum proteome pattern outperformed available biomarkers analyzed in blood by immunoassays.

## Methods

### Characteristics of patient and control groups

The clinical part of the study was carried out at the Maria Sklodowska-Curie Memorial Cancer Center and Institute of Oncology, Gliwice Branch, between May 2006 and January 2008. Ninety-two patients diagnosed with clinical stage I or II breast cancer were included in the study, of average age 58.5 years (range 31–74 years). Patients were classified according to the TNM scale; the majority were scored as T1 and T2 (47% and 45%, respectively) as well as N0 and N1 (75% and 24%, respectively), and none had diagnosed metastases (all M0). Biopsy material was used to assess for histopathological tumor grade (27% G1, 45% G2, 28% G3), as well as for expression of estrogen receptor (63% ER+) and progesterone receptor (60% PR+) by immunohistochemistry. Serum samples were collected before the start of therapy. One hundred and four female volunteers were included as a control group; they were required to be free of any known acute or chronic illness and were not treated with any anticancer therapy in the past. The average age in this group was 54 years (range 32–77 years). The study was approved by the appropriate Ethics Committee and all participants provided informed consent indicating their voluntary participation.

### Preparation of serum samples

Samples were collected and processed following a standardized protocol. Blood was collected in a 5 ml Vacutainer Tube (Becton Dickinson), incubated for 30 min. at room temperature to allow clotting, and then centrifuged at 1000 g for 10 min. to remove the clot. The serum was aliquoted and stored at -70°C. Directly before analysis, samples were diluted 1:5 with 20% acetonitrile (ACN) in water, then applied onto an Amicon Ultra-4 membrane (50 kDa cut-off) in a spin column and centrifuged at 3000 g for 30 min. This removed the majority (up to 80%) of albumin and other abundant high-molecular weight proteins from the serum samples (not shown).

### Mass spectrometry

Samples were analyzed using an Autoflex MALDI-ToF mass spectrometer (Bruker Daltonics, Bremen, Germany); the analyzer worked in the linear mode and positive ions were recorded in the mass range between 2,000–10,000 Da. Mass calibration was performed after every four samples using standards in the range of 5000 to 17,500 Da (Protein Calibration Standard I, Bruker Daltonics). Prior to analysis each sample was loaded onto a ZipTip C18 tip-microcolumn by passing it through repeatedly 10 times, column was washed with water and then eluted with 1 μl of matrix solution (30 mg/ml sinapinic acid in 50% ACN/H_2_O and 0.1% TFA with addition of 1 mM n-octyl glucopyranoside) directly onto the 600 μm AnchorChip (Bruker Daltonics) plate. ZipTip extraction/loading was repeated twice for each sample and for each spot on the plate two spectra were acquired after 120 laser shots (i.e. four spectra were recorded for each sample). Spectra were exported from the Bruker FlexAnalysis 2.2 software in standard 8-bit binary ASCII format; they consisted of approximately 45,400 measurement points describing mass to charge ratios (m/z) for consecutive [M+H]^+ ^ions and the corresponding signal abundances, covering the range of analyzed m/z values.

### Analysis of protein tumor markers in plasma

Plasma samples were obtained after centrifugation of blood on a Ficoll gradient (Lymphoprep™, ICN), and then levels of selected markers were quantified using standard methods of immuno-diagnostics. Enzyme-Linked Immunosorbent Assay (ELISA) was used for assessment of leptin (DRG Diagnostics) and osteopontin (R&D Systems), Chemiluminescent Microparticle Immunoassay (CMIA) for assessment of CEA (Abbott), Trace Resolved Amplified Cryptate Emission (TRACE) for assessment of CYFRA 21.1 (Brahms), and Microparticle Enzyme Immunoassay (MEIA) for assessment of CA15.3 (Abbott). In addition, the level of osteopontin was analyzed in serum samples as described above.

### Data Processing and Statistical Analysis

The preprocessing of data that included averaging of technical repeats, interpolation of missing or non-aligned points, binning of neighboring points to reduce data complexity, removal of the spectral area below baseline and the total ion current (TIC) normalization was performed according to procedures considering to be standard in the field [45,46]. In the second step the spectral components, which reflected [M+H]^+ ^ions recorded at defined m/z values, were identified using decomposition of mass spectra into their Gaussian components. The spectra were modeled as a sum of Gaussian bell-shaped curves, then models were fitted to the experimental data by a variant of the expectation maximization (EM) algorithm [47]. In a few cases when the standard deviation of a Gaussian exceeded a value of 50 the corresponding spectral component was excluded from further more detailed analyses. Based on the decomposition of the average mass spectrum into the Gaussian components, the classifier features were computed by the scalar product with the Gaussian curves treated as kernel functions. The classification used version of the Support Vector Machine (SVM) algorithm described by Schölkopf and coworkers [48]. The size of the training sample was changed from 20% to 90% of the whole dataset, and for each size the two-step training/validation procedure was repeated 1000 times to estimate the average error rate and its 95% confidence interval, which characterized the accuracy of classification. In order to further characterize the quality of classification, receiver operating curves (ROC) were computed by changing the value of the classification threshold in the SVM classifiers, and averaging the obtained specificity/sensitivity proportions over 1000 random validation experiments. We tested the performance of classification with classifiers built of different numbers of spectral components by estimating the level of total errors, as well the number of false positive and false negative classifications. Construction and validation of a classifier is a statistical process, i.e. many different classifiers built of a given number of spectral components were tested (1000 random splits of the dataset), and those which pass the quality threshold could be built of different spectral components. Thus, to identify the components that are the best determinants of a specific proteome pattern we looked for the most frequent components in classifiers that correctly classified samples. The performance of classifiers built of optimized components was assessed by standard logistic regression (1000 iterations with a 50/50 split of the training/validation set).

## Results and discussion

### Classifiers built on spectral components that determine proteome patterns

The low-molecular-weight fraction of the blood serum proteome consists of numerous peptides, proteins and their fragments. Some of these interact with each other, and a substantial fraction of this blood proteome compartment is carried by albumin as cargo peptides [49,50]. For this reason we implemented dilution of serum samples with a denaturing organic solvent (acetonitrile) that destroyed the majority of protein interactions and allowed analysis of individual peptides dissociated from (not interacting with) other proteins (e.g., albumin). Characteristic features of MALDI ionization are that most ions created during laser irradiation are singly charged (multiply charged ions, especially those with low m/z values, have very low abundances and can be are neglected), and that these ions are not fragmented under the ionization conditions applied. In other words, peaks registered in a MALDI mass spectrum correspond to mono-protonated peptide/protein molecular ions [M+H]^+ ^described by m/z values that reflect actual molecular weights increased by the mass of the proton. However, when MALDI mass spectra are recorded over a wide range of m/z values (like the 2–10 kDa range in this study) the expected mass accuracy is relatively low and reaches 0.01–0.1% of the analyte's molecular mass, which corresponds to a few Daltons in the range of m/z values analyzed. In consequence, the relative broadening of spectral peaks recorded for the [M+H]^+ ^ions could reflect the low resolution of the analyzer operating in the linear mode or might result in overlapping of ions originating from protein/peptides of very similar molecular masses. In addition, because of technological imperfections there might be some shift in the positions of peptide ions between measurements, which adds more complexity to analyses of large datasets. For this reason, some approaches used for analysis of large datasets relay on alignment of identified spectral peaks [45], which requires numerical "stretching" of spectra before further analyses.

Here we decided to implement an original mathematical procedure based on modeling average spectra and then fitting actual experimental spectra into such a model. Averaging was performed over either the whole dataset or data for cancer patients only, depending on whether the model was used to discriminate cancer and normal samples or different clinical outcomes of patients. We tested models with different numbers of components, and found that for the mass spectra analyzed in the present work 300 components ensured both sufficient fidelity of the model and its efficient computation (not shown). As a result of computation an "average" spectrum was decomposed into spectral components characterized by the exact molecular weight (m/z values of recorded [M+H]^+ ^ions) and the interval where fit corresponding peaks in at least 95% of actual spectra expected in the dataset (+/-95% CI). The resulting spectral components reflect peaks recorded in multiple samples during mass spectrometric analysis, which contained either single peptide/protein ions or a combination of a few ions of very similar m/z values. This approach allowed us to avoid artifacts resulting from the peak alignment and facilitated quantitative analysis of data by simple assessment of signal volumes that fitted to a given component within its 95% CI. Having identified and quantified spectral components, one could find certain whose abundances were significantly different between groups of samples (e.g. between cancer patient and healthy samples) which could be defined as "differentiating". However, to obtain more reliable classification of samples we used spectral components to build multi-component classifiers that determined proteome patterns characteristic for groups, and looked for the most frequent components in classifiers that classified samples correctly.

### Identification of components that determine proteome patterns specific for healthy persons and breast cancer patients

**Figure 1:**
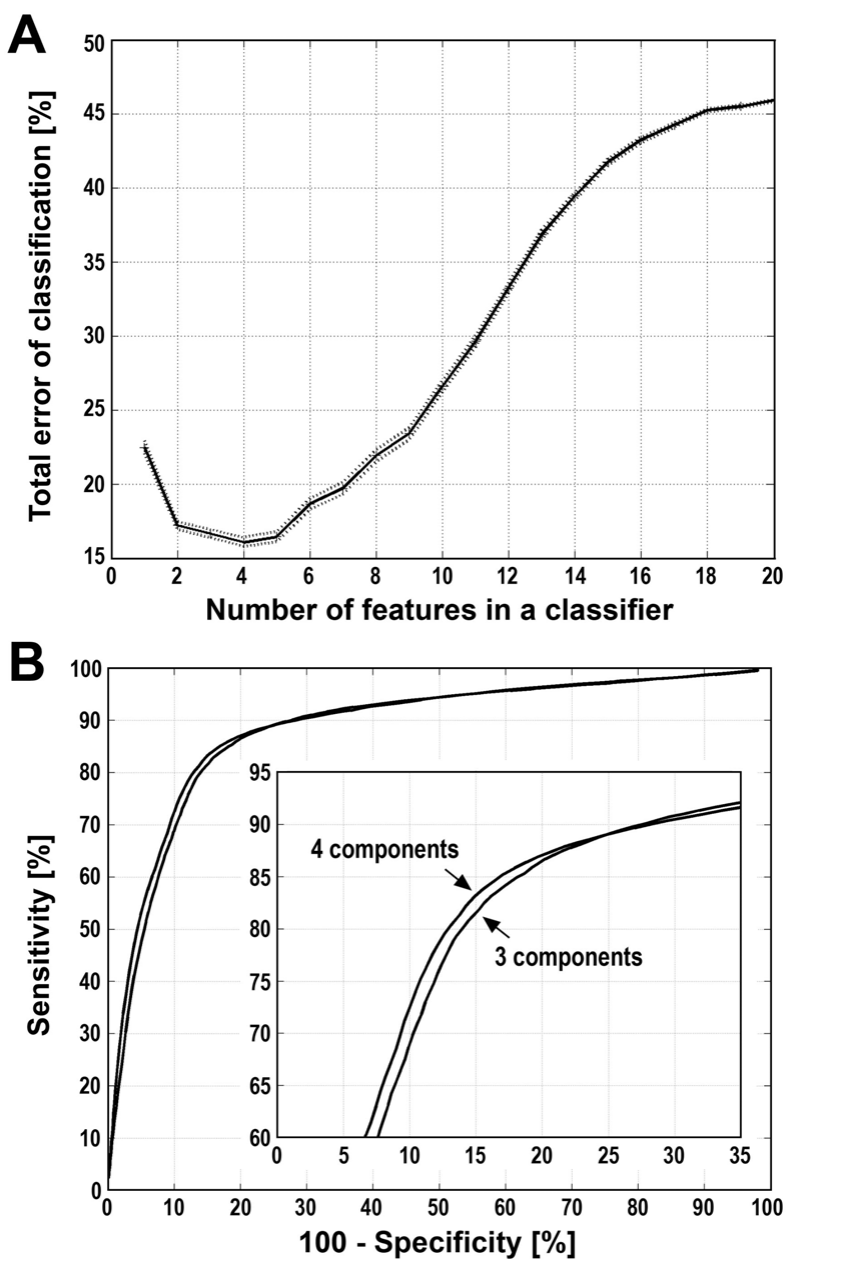
**Estimation of the performance of classification of breast cancer samples**. **A **– The total error rate was plotted against the number of features (i.e. spectral components) in the classifier. Shown are average error rates and 95% confidence intervals calculated based on 1000 random validation experiments with 50:50 training/validation split of data. **B **– Estimation of the sensitivity and specificity of the classification for classifiers built of three or four spectral components. The ROC curve was computed by changing the value of the probability threshold in the SVM classifier from 0.0 to 1.0, and averaging the specificity obtained versus sensitivity rate over 1000 random repeats of training and validation.

At first we compared the serum proteome patterns of 104 healthy women and 92 early stage breast cancer patients. Spectral components corresponding to protein/peptide [M+H]^+ ^ions recorded in MALDI mass spectra were used to built classifiers to perform cancer/healthy control classifications as described above. The best classification performance was obtained with classifiers built of 2–5 features, i.e. spectral components (Fig. 1A). To estimate the sensitivity and specificity of classification, ROC curves [51] were computed for classifiers built of 3 or 4 spectral components. According to our estimations these classifiers allowed classification of cancer patients with 85% specificity and 82–83% sensitivity (Fig. 1B).

**Figure 2:**
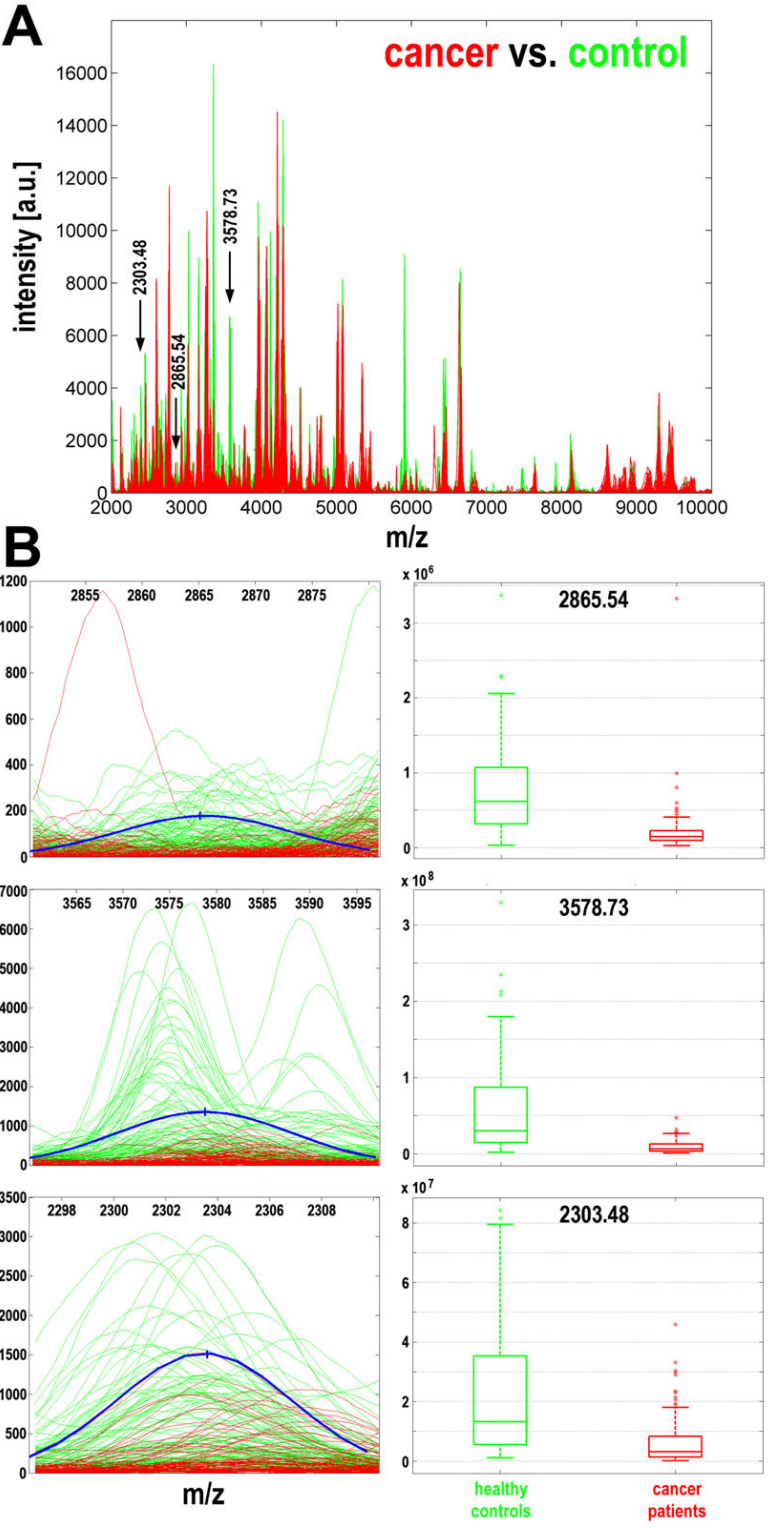
**Characterization of spectral components essential for cancer classification**. **A **– The three most frequent differentiating components are marked with arrows along the mass spectra of serum samples of cancer patients (red lines) and healthy controls (green lines). **B **– Actual spectral plots of three selected components for cancer patients (red lines) and healthy controls (green lines), as well as modeled Gaussian kernels (blue curves); X-axes represent the m/z values, Y-axes represent intensities. Box-plots on the right represent quantification of the abundance of spectral components in samples from cancer patients (red) and healthy controls (green) (shown are minimum, lower quartile, median, upper quartile and maximum values; outliers are marked by asterisks).

In further analyses we looked for the most frequent spectral components in classifiers that correctly classified breast cancer samples. The three most important components corresponded to the following [M+H]^+ ^peptide ions: m/z = 2865.54, m/z = 3578.73, and m/z = 2303.48 (Fig. 2A). Most interestingly, two of these (m/z = 2865.54 and m/z = 3578.73) were present in nearly all well-performing classifiers, while the third (m/z = 2303.48) was present in 78% of classifiers; it was noteworthy that all other spectral components appeared in classifiers less frequently (<50%; Table 1). Importantly, these most frequent components of cancer classifiers had very high potency to differentiate control and cancer samples by themselves; the statistical significance of differences obtained in univariant analyses for these three peaks were at the level of p-values from 10^-20 ^to 10^-14 ^(they remained highly significant after application of the Bonferroni correction for multiple testing; Table 1). Fig. 2B shows fragments of mass spectra in the near vicinity of the components that were the most frequent features of these breast cancer classifiers; the actual spectral lines for samples from all 196 individuals are shown together with the model component. The levels of such components in samples from individual breast cancer patients and healthy controls were quantified and are shown as box-plots (Fig. 2B).

**Table 1:** Characteristics of spectral components that differentiated samples from breast cancer patients and healthy controls.

Component m/z value	-95% CI	+ 95% CI	S.D.	p-value	Corrected p-value	Frequency	Change
2294.67	2283.38	2305.96	5.76	1.28e-12	3.84e-10	46%	D

**2303,48**	2296,88	2310,09	3,37	**6.25e-14**	**1.88e-11**	**78%**	D

2554.37	2540.32	2568.41	7.16	4.13e-07	1.24e-04	1%	U

2845.58	2838.34	2852.81	3.69	3.59e-12	1.08e-09	21%	D

**2865.54**	2864.46	2866.62	7.73	**4.19e-20**	**1.26e-17**	**100%**	D

3283.73	3265.34	3302.13	9.39	6.60e-07	1.98e-04	1%	U

3360.19	3352.06	3368.31	4.15	5.69e-11	1.71e-08	22%	D

3427.46	3401.71	3453.21	13.14	8.11e-11	2.43e-08	7%	D

**3578.73**	3577.42	3580.04	9.36	**5.84e-18**	**1.75e-15**	**99%**	D

3874.18	3863.89	3884.47	5.25	8.08e-09	2.42e-06	3%	D

3895.05	3882.03	3908.06	6.64	1.58e-11	4.74e-09	6%	D

4965.77	4945.35	4986.19	10.42	1.91e-08	5.73e-06	5%	D

6061.80	6050.15	6073.45	5.94	9.53e-09	2.86e-06	5%	D

6743.99	6734.13	6753.85	5.03	2.99e-08	8.97e-06	2%	D

Shown are the most frequent spectral components (m/z values), their 95% confidence intervals, standard deviations of the corresponding model Gaussians, and relative frequencies in cancer classifiers built of 4 features. The p-values are for differences between patients and healthy controls measured by the Mann-Whitney U test for each individual component (also shown after the Bonferroni correction against multiple testing). The change refers to either increased (U) or decreased (D) abundance of a given peptide in cancer samples compared to control samples. The three most frequent components are underlined.

We also found that 49 out of 300 modeled spectral components (i.e., 16%) had themselves a high potential to differentiate control and cancer samples in univariant analyses (p-value < 0,05 after the Bonferroni correction). Furthermore, all 14 spectral components that appeared in at least 1% of classifiers built of 4 features retained a very high differentiation potential in univariant analyses (p-value < 0.0002 after the Bonferroni correction; Table 1). In addition, we cross-compared spectral components that showed some differentiating power in our study (90 spectral components with uncorrected p-value < 0.005) with spectral peaks that were reported in some other published studies to differentiate breast cancer from healthy control samples (uncorrected p-value < 0.005). The correspondence of [M+H]^+ ^ions was based on ± 0.2% of the m/z values. We found that at least 15 of these spectral components had a corresponding differentiating peak in comparable studies (although not always showing the same tendency; Table 2). This reproducibility, observed in spite of large differences in experimental and computational design, indicates a potency of convergence toward a common proteome pattern specific for breast cancer samples. Interestingly, two spectral components that appeared the most important for cancer classification in our study (i.e., m/z = 2865.54 and m/z = 3578.73) were not reported as differentiating peaks in other studies. We note, however, that in our study serum was analyzed after removal of albumin and components bound to it, which apparently influenced the pattern of mass spectra of the low-molecular-weight fraction of the blood proteome. We observed markedly increased levels of some spectral components in albumin-depleted samples as compared to those analyzed directly (not shown), which could possibly be explained by a reduced efficiency of ionization and detection of certain less abundant peptides in the presence of albumin [49].

**Table 2:** Comparison of discriminating spectral components/peptide peaks found in this study and in other published work.

This study			Other studies					
	
m/z value	p-value	Change	m/z value	p-value	Change	Ref.	Study design	Identity
	
2303.48	6.25e-14	D	2306.20	1.09e-06	U	35	MALDI/serum/A	C4a
	
2356.91	2.47e-04	D	2359.09	4.07e-12	U	35	MALDI/serum/A	ITIH4
	
2378.80	8.91e-06	D	2380.03	1.26e-07	U	35	MALDI/serum/A	Fibrinogen
	
2510.80	4.65e-08	D	2509.16	5.56e-13	U	35	MALDI/serum/A	ApoA-IV
	
2599.75	6.03e-04	U	2603.15	2.08e-07	U	35	MALDI/serum/A	Factor XIIIa
	
3020.51	5.49e-03	U	3017.85	1.50e-03	U	43	SELDI/NAF/M	
	
3273.96	1.08e-03	U	3278.71	1.05e-05	D	42	SELDI/serum/M	
	
3283.73	6.80e-07	U	3281.5	1.77e-04	U	38	MALDI/serum/M	
	
3973.35	1.51e-06	D	3284.74	3.00e-04	U	43	SELDI/NAF/M	
	
4648.09	3.48e-07	U	3975.99	3.06e-05	D	42	SELDI/serum/M	
	
5105.44	4.66e-03	U	4648	4.13e-03	D	42	SELDI/serum/M	
	
6802.40	1.42e-03	D	5101.8	4.90e-03	U	43	SELDI/NAF/M	
	
8116.60	3.41e-04	D	6807.26	1.90e-03	D	42	SELDI/serum/M	
	
8134.75	9.61e-04	D	8116	1.00e-06	U	29,33	SELDI/serum/M	C3a
	
8656.46	2.73e-04	U	8138.56	7.89e-07	U	42	SELDI/serum/M	
	
			8657.2	1.00e-03	U	37	SELDI/Serum/E	

Uncorrected p-values are based on Mann-Whitney U tests in this study. Correspondence of peptide peaks is based on a difference of less than ± 0.2% of the m/z values of [M+H]^+ ^ions. The column "Change" refers to an increased (U) or decreased (D) abundance of a given peptide in breast cancer samples comparing to control samples, and the column "Identity" shows the protein from which the corresponding fragment is derived. Corresponding peptide peaks were found in six studies based on either MALDI or SELDI spectrometry; patient groups consisted of either early (E), advanced (A) or mixed (M) stages. One study analyzed the nipple aspirate fluid (NAF).

### Serum proteome patterns identified by MALDI-ToF analyses are similar for different sub-groups of early stage breast cancer patients

**Figure 3:**
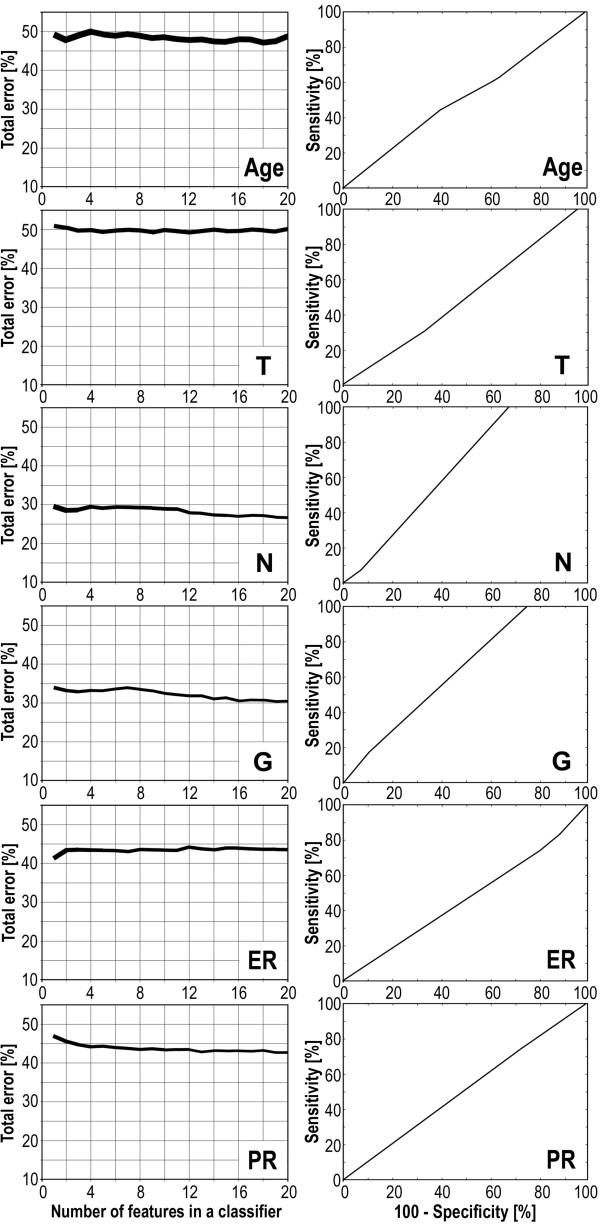
**Estimation of differences of serum proteome patterns between sub-groups of breast cancers patients**. Patients were differentiated by age, primary tumor size (T), lymph nodal status (N), histopathological grade (G), and estrogen (ER) and progesterone (PR) receptor expression. **A **– The total error rates of classification plotted against the number of features in the classifiers as in Fig. 1A; the actual line width corresponded to 95% confidence intervals. **B **– ROC curves computed for classifiers built of 15 spectral components for each comparison (computation was done as described in Fig. 1B).

Having established that MALDI-ToF analysis of serum peptides identified proteome patterns characteristic for cancer patients, we next examined whether features of peptide profiles would differentiate specific subgroups of patients. First, the group of patients was divided into two equal subgroups according to their age (younger or older then 56.5 years, which was the median), and then spectral classifiers were built according to the methodology described above. In this particular case the performance of classification was about 50% independently of the number of spectral components (features) in classifiers (Fig. 3A), and consequently the classifier had about 50% specificity and 50% sensitivity as shown on the corresponding ROC curve (Fig. 3B). This indicated that there was no real difference in serum proteome patterns between subgroups of patients divided according to their age. This result could be expected because in the whole group there was only 1 patient younger then 35 years which is normally considered an early appearance of cancer, and thus our two age-related subgroups most possibly reflect a random division of the group. Having this "negative control" classification, we next aimed to identify serum proteome patterns specific for subgroups of patients with different clinical and molecular outcomes. We compared patients with different primary tumor size (T1 vs. T2), lymph node status (N0 vs. N1), histopathological grade (G1 and G2 vs. poorly differentiated G3), and also two well-established breast cancer prognostic and predictive molecular markers, expression of estrogen receptor or progesterone receptor [rev. in: [52-54]]. For each comparison the performance of classification (total error of classifiers built of 1 to 20 features) and the corresponding ROC curves for classifiers built of 15 spectral components (these were representative of ROC curves computed for classifiers built of 1 to 20 features) are shown in Fig. 3. Most importantly, we observed a low performance of putative classification with a high level of errors for all analyses carried out. Although analyses based on the nodal status and the histopathological grade showed relatively moderate levels of total error (Fig. 3A), they had a very high level of false negative classifications (not shown) which was related to the unbalanced number of subgroups compared (see Table 3); the shape of the corresponding ROC curves also reflect this unbalance (Fig. 3B).

**Table 3:** Comparison of serum proteome patterns among different sub-groups of breast cancer patients.

**Component** **m/z value**	**S.D.**	**p-value**	**Frequency [%]**
**Age **(median = 56.5 years)		**>median **(n = 42) **vs. <median **(n = 42)	

5353.64	11.54	0.020	48.4

2475.96	2.37	0.029	19.9

4098.35	25.90	0.032	22.5

3024.45	11.71	0.035	20.2

4070.43	5.58	0.048	19.2

**T – primary tumor size**		**T1 **(n = 44)**vs. T2 **(n = 40)	

5353.64	11.54	0.033	21.9

2873.93	7.13	0.038	20.5

5343.98	6.26	0.051	15.8

3024.45	11.71	0.073	32.8

3249.64	5.24	0.075	12.4

**N – nodal status**		**N0 **(n = 63)**vs. N1 **(n = 20)	

8618.25	10.83	0.024	45.1

8602.25	29.35	0.036	29.5

2909.04	10.58	0.038	32.5

8607.98	5.60	0.040	25.7

8682.92	8.83	0.047	28.2

**G – histopathological grade**		**G1+G2 **(n = 54)**vs. G3 **(n = 20)	

2937.50	6.76	0.004	34.1

2556.63	8.07	0.007	44.1

2909.04	10.58	0.011	36.8

7547.58	12.44	0.022	37.7

4793.63	5.91	0.026	8.1

**ER – estrogen receptor status**		**ER(-) **(n = 29) **vs. ER(+) **(n = 51)	

7915.93	29.15	0.038	42.4

6302.67	4.03	0.039	23.6

8246.58	14.13	0.043	27.5

2599.96	3.93	0.044	28.9

3367.65	13.43	0.061	22.4

**PR – progesterone receptor status**		**ER(-) **(n = 32) **vs. ER(+) **(n = 49)	

7101.57	8.39	0.002	50.7

9965.62	16.77	0.014	37.3

7750.49	24.72	0.015	30.6

3367.65	13.43	0.018	30.5

9934.38	23.21	0.020	28.3

The five spectral components with the lowest p-values were selected for each comparison. Shown are spectral components (m/z values), S.Ds. of the corresponding model Gaussians, and their relative frequencies in classifiers. The p-values (uncorrected) are for differences measured by the Mann-Whitney U test for each individual component.

The spectral components identified by Gaussian model decomposition were also used for univariant analyses of differences between the subgroups described above. Table 3 presents examples of the top five spectral components with the lowest p-values identified for each of such comparisons. Most importantly, although in standard analyses the levels of some components were different between the subgroups compared, none of these differences appeared significant after application of the Bonferroni test for multiple testing correction (not shown). This result was in complete agreement with results of classification by multi-component classifiers (Fig. 3), which clearly showed similar serum proteome patterns identified by MALDI-ToF analyses in different sub-groups of the early stage breast cancer group. This finding suggested that the multi-component cancer classifier described above might be potentially applicable for early detection of breast cancer, independent of further more detailed clinical and pathological features.

### A classifier built on MS-based serum proteome pattern outperforms available protein biomarkers analyzed in blood by immunoassays

To further assess potential diagnostic power of multi-component classifier described above we compared reliability of classification based on biomarker identified by mass spectrometry with the one that based on available protein biomarkers analyzed in blood by immunoassays. Five markers were selected: carcinoembryonic antigen (CEA), carbohydrate antigen CA15.3, cytokeratin fragment CYFRA-21.1, leptin and osteopontin, which had putative diagnostic value for breast cancer, especially at advanced clinical stages, yet none of them was routinely used for early diagnostics of breast cancer [55-60]. The plasma levels of these biomarkers were quantified in a group of early stage breast cancer patients (which largely overlapped with the group examined using MALDI-ToF mass spectrometry) and compared with corresponding levels in a group of healthy donors (Table 4). We observed that the level of osteopontin was markedly increased in plasma of cancer patients, and the difference had a high level of statistical significance (p = 0.0003). The differences were much less significant for the four other markers, and therefore osteopontin alone was used in further analyses. The anti-osteopontin antibody used for ELISA recognized all four isoforms (OPN-a, OPN-b, OPN-c, OPN-d) and their different proteolytic fragments present in blood, and thus direct correlation of the ELISA results with MALDI-ToF analyses was not possible. When the plasma level of osteopontin was used for cancer classification it showed 88% sensitivity but only 28% specificity (as tested by the standard logistic regression method).

**Table 4:** Levels of tumor markers in plasma of breast cancer patients and healthy controls.

**Group**	**n**	**Median**	**Mean**	**S.D.**	**Lower-upper quartile**	**p-value**
**CEA **(ng/ml)						

healthy controls	58	1.13	1.62	1.46	0.84 – 1.75	0.04
	
cancer patients	37	1.54	2.45	3.11	1.00 – 2.11	

**CA15-3 **(U/ml)						

healthy controls	58	12.3	13.28	5.45	9.5 – 16.4	0.63
	
cancer patients	37	14.0	13.74	5.79	8.3 – 18.5	

**CYFRA 21.1 **(ng/ml)						

healthy controls	58	0.41	0.53	0.48	0.24 – 0.60	0.06
	
cancer patients	37	0.54	0.63	0.44	0.35 – 0.75	

**Leptin **(ng/ml)						

healthy controls	58	27.70	33.51	23.01	17.80 – 41.80	0.05
	
cancer patients	37	23.01	24.19	16.09	10.02 – 31.11	

**Osteopontin **(ng/ml)						

healthy controls	50	45.90	47.13	11.9	38.70 – 52.20	0.0003
	
cancer patients	73	54.73	59.47	15.37	47.13 – 66.98	

Shown are median, mean and S.D. values, as well as lower and upper quartiles. The p-values are for differences between patients and healthy controls measured by the Kruskal-Wallis test.

With the aim of constructing a putative marker useful in early diagnosis of breast cancer, we decided to combine features of the serum proteome pattern identified by MALDI-ToF MS analysis and the level of osteopontin measured by ELISA. Three spectral components, m/z = 2865.54, m/z = 3578.73, and m/z = 2303.48 Da, which were the most frequent components of the cancer classifier described above, were selected for these analyses. The marker built of this three spectral components showed 78% specificity and 88% sensitivity when tested by the standard logistic regression method. Then, the level of osteopontin was re-tested in serum samples from the cancer patients and healthy individuals subjected to the MS-based study. In this case, however, the average concentration of osteopontin in serum was about two-fold lower as compared to that in plasma, and the difference between cancer patients and healthy persons was much less pronounced. The biomarker built of the serum level of osteopontin alone showed 84% specificity and but only 12% sensitivity when tested by the standard logistic regression method. Finally we tested the performance of a marker built of four features, the three most frequent spectral components (m/z = 2303.48, 2865.54, and 3578,73) and osteopontin. This combined marker showed 78% specificity and 88% sensitivity, the same as the marker built of three spectral peaks alone.

## Conclusion

Here we confirmed the high potential of serum proteome pattern analysis by MALDI-ToF spectrometry for the differentiation between early breast cancer patients and healthy controls. Most importantly, a classifier built on this analysis outperforms those based on available protein biomarkers analyzed by immunoassays in blood. However, further combination of MS-based serum proteome pattern analysis with traditional cancer markers might possibly result in a biomarker with a reliability high enough for practical implementation in the early detection and diagnostics of breast cancer.

## Competing interests

The authors declare that they have no competing interests.

## Authors' contributions

MP – performed experiments, interpreted results, LM – performed experiments, interpreted results, JP – performed mathematical modeling and statistical analyses, KB – collected and interpreted clinical data, EN – collected and interpreted clinical data, AW – performed experiments, AC – performed immunoassyas, RD – performed immunoassays, MS – designed and interpreted MS data, drafted manuscript, AP – designed mathematical modeling, drafted manuscript, RT – designed and interpreted clinical part of the study, drafted manuscript, PW – designed and interpreted experiment, prepared final manuscript. All authors read and approved the final manuscript.
